# IRE1α protects against osteoarthritis by regulating progranulin-dependent XBP1 splicing and collagen homeostasis

**DOI:** 10.1038/s12276-023-01106-w

**Published:** 2023-11-01

**Authors:** Li Liang, Fengmei Zhang, Naibo Feng, Biao Kuang, Mengtian Fan, Cheng Chen, Yiming Pan, Pengfei Zhou, Nana Geng, Xingyue Li, Menglin Xian, Lin Deng, Xiaoli Li, Liang Kuang, Fengtao Luo, Qiaoyan Tan, Yangli Xie, Fengjin Guo

**Affiliations:** 1 Laboratory of Developmental Biology, Department of Cell Biology and Genetics, School of Basic Medical Sciences, Chongqing Medical University, 400016 Chongqing, China; 2 Laboratory Animal Center, Chongqing Medical University, 400016 Chongqing, China; 3 Department of Orthopedics, The 2nd Affiliated Hospital of Chongqing Medical University, 400072 Chongqing, China; 4 Department of Orthopedics, The 1st Affiliated Hospital of Chongqing Medical University, 400016 Chongqing, China; 5 Chongqing Key Laboratory of Oral Diseases and Biomedical Sciences, College of Stomatology, Chongqing Medical University, 400016 Chongqing, China; 6 Department of Wound Repair and Rehabilitation Medicine, Center of Bone Metabolism and Repair (CBMR), State Key Laboratory of Trauma and Chemical Poisoning, Research Institute of Surgery, Daping Hospital, Army Medical University, 400042 Chongqing, China

**Keywords:** Bone, Cell biology

## Abstract

Osteoarthritis (OA) is a full-joint, multifactorial, degenerative and inflammatory disease that seriously affects the quality of life of patients due to its disabling and pain-causing properties. ER stress has been reported to be closely related to the progression of OA. The inositol-requiring enzyme 1α/X-box-binding protein-1 spliced (IRE1α/XBP1s) pathway, which is highly expressed in the chondrocytes of OA patients, promotes the degradation and refolding of abnormal proteins during ER stress and maintains the stability of the ER environment of chondrocytes, but its function and the underlying mechanisms of how it contributes to the progression of OA remain unclear. This study investigates the role of IRE1α/*ERN1* in OA. Specific deficiency of *ERN1* in chondrocytes spontaneously resulted in OA-like cartilage destruction and accelerated OA progression in a surgically induced arthritis model. Local delivery of Ad*ERN1* relieved degradation of the cartilage matrix and prevented OA development in an ACLT-mediated model. Mechanistically, progranulin (PGRN), an intracellular chaperone, binds to IRE1α, promoting its phosphorylation and splicing of XBP1u to generate XBP1s. XBP1s protects articular cartilage through TNF-α/ERK1/2 signaling and further maintains collagen homeostasis by regulating type II collagen expression. The chondroprotective effect of IRE1α/*ERN1* is dependent on PGRN and XBP1s splicing. *ERN1* deficiency accelerated cartilage degeneration in OA by reducing PGRN expression and XBP1s splicing, subsequently decreasing collagen II expression and triggering collagen structural abnormalities and an imbalance in collagen homeostasis. This study provides new insights into OA pathogenesis and the UPR and suggests that IRE1α/*ERN1* may serve as a potential target for the treatment of joint degenerative diseases, including OA.

## Guardians of cartilage: IRE1α shields against osteoarthritis through progranulin

Researchers have discovered that the *ERN1*/IRE1α plays a protective role in cartilage by regulating the PGRN-dependent XBP1 splicing and collagen homeostasis. The study found that cartilage-specific deficiency of *ERN1* in mice led to spontaneous osteoarthritis development and more severe cartilage damage in a surgically induced arthritis model. Furthermore, the chondroprotective effect of IRE1α was dependent on PGRN, an intracellular molecular chaperone that interacts with *ERN1*/IRE1α. This research provides new insights into the role of IRE1α in osteoarthritis progression and highlights its potential as a therapeutic target for degenerative joint diseases.

## Introduction

Osteoarthritis refers to a group of chronic degenerative bone diseases involving various tissues, including articular cartilage, subchondral bone, and synovial tissue around the joint^1–3^. Under normal physiological conditions, the degradation and synthesis of cartilage components maintain a dynamic balance. Any damage and degeneration of articular cartilage causes an imbalance in cartilage metabolism, leading to progressive tissue loss and dysfunction^4,5^. The cartilage extracellular matrix (ECM) is mainly composed of collagen fibers, proteoglycans and glycosaminoglycans. The accumulation of misfolded and unfolded ECM proteins, such as collagen types II, IX, X and XI and aggrecan, can influence growth plate development and cause cartilage-related diseases, including chondrodysplasia^6^. The synthesis of these ECM mutant proteins can trigger endoplasmic reticulum (ER) stress to induce an unfolded protein response (UPR), which leads to the activation of canonical ER stress sensors, including inositol-requiring enzyme-1α (IRE1α), PERK, and ATF6^7^. The assembly and folding of secreted proteins in the ER are complex and exquisitely regulated processes that maintain ER homeostasis. Stable ER stress and UPR signals and effects are essential for normal chondrogenic differentiation and hypertrophic maturation^8^.

Human IRE1α, encoded by the endoplasmic reticulum to nucleus signaling 1 (*ERN1*) gene, is equipped with a serine/threonine protein kinase domain and a ribonuclease domain and participates in regulating the folding of proteins and maintaining ER homeostasis^9^. IRE1α is closely associated with cartilage or bone growth and development. Tohmonda et al. reported that specific inactivation of IRE1α in hematopoietic stem cells leads to increased bone mass and decreased osteoclast number in vivo^10^. Cameron et al. analyzed the role of the IRE1α/XBP1 pathway in the pathology of Schmid-type metaphyseal achondroplasia (MCDS) and proposed that the pathology of MCDS is supported by XBP1-independent UPR-induced dysregulation of C/EBP-β-mediated chondrocyte differentiation^11^. Tohmonda et al. proposed that the IRE1α-XBP1 signaling pathway is involved in osteoblast differentiation by facilitating the transcription of osterix^12^. Furthermore, myofiber-specific defects in IRE1α weakened skeletal muscle regeneration in adult mice^13^ and activated ER stress, and the UPR signaling pathway component IRE1α in the body’s immune cells can increase the release of inflammatory cytokines and further promote the production of inflammation^14^. We previously reported that IRE1α enhances chondrocyte viability and inhibits cell apoptosis in chondrocytes, whereas IRE1α deficiency increases the apoptosis of chondrocytes by upregulating the proapoptotic factors caspase 3, pJNK1, and CHOP^15^. We also designed and constructed a novel si*ERN1*-nanoprodrug with universal biocompatibility, long-term drug release responsiveness, superior targeting properties, and strong anti-inflammatory therapeutic effects in mouse collagen-induced arthritis and inflammatory bowel disease models^16^. Although some studies have reported the potential efficacy of IRE1α regulation in skeletal development and related diseases, as Huang noted, IRE1α plays an important role in chondrocytes, and the understanding of IRE1α responses and cell death fate remains controversial^17^. Given the uncertainties in the regulation of cartilage function by IRE1α and the remarkable complexity and heterogeneity exhibited by osteoarthritis, it is important to elaborate on the mechanism of *ERN1*/IRE1α in the occurrence and development of osteoarthritis.

In the current study, we not only elucidated the role of *ERN1*/IRE1α in OA but also revealed the mechanism by which *ERN1* deficiency leads to the structural abnormality of collagen through PGRN and XBP1 splicing and proposed a possible functional compensatory mechanism in the absence of *ERN1*. This study provides a new strategy for the precise treatment of clinical OA.

## Materials and methods

### Human subjects

All biomedical studies involving humans in this study were reviewed and approved by the Ethics Committee of Chongqing Medical University, and full written consent was obtained before the operative procedure. In this study, 12 clinical samples were collected and used for Western blotting and RT‒qPCR: 3 normal and 9 osteoarthritic cartilage tissues. All samples were stored in liquid nitrogen for a long time.

### Mice

All animal studies were performed in accordance with institutional guidelines and approval by the Ethics Committee of Chongqing Medical University. *ERN1*^flox/+^ mice were purchased from Shanghai Biomodel Organism Science & Technology Development Co., Ltd (Shanghai, China). *Col2a1*-Cre mice on a C57BL6/J background were gifted by Dr. Lin Chen (Army Medical University, Chongqing, China). *ERN1*^flox/flox^, *Col2a1*-Cre mice (*ERN1*cKO) and *ERN1*^flox/flox^ mice (Cre-negative control mice) were obtained in accordance with previously described methods^1,2^. *GRN*^−/−^ mice were purchased from the Jackson Laboratory.

### Cell culture

The human chondrocyte cell line C28/I2 was kindly provided by Professor Liu Chuanju (New York University, USA). RAW 264.7 cells were purchased from the Cell Bank of the Chinese Academy of Sciences (Shanghai, China). All cells were routinely assessed every 2 months for mycoplasma contamination using the EZ-PCR mycoplasma test kit (Biological Industries, Israel) according to the manufacturer’s protocol. C28/I2 cells, HEK 293 cells and 293T cells were maintained in Dulbecco’s modified Eagle’s medium (DMEM) with 100 units/ml streptomycin and penicillin and 10% fetal bovine serum (FBS). RAW 264.7 cells were cultured in Dulbecco’s modified Eagle’s medium alpha (α-MEM) containing 10% FBS and 100 units/ml streptomycin and penicillin. All cells were cultured at 37 °C in a humidified incubator with 5% CO_2_.

### Primary mouse chondrocytes

Immature murine articular chondrocytes were isolated by enzymatic digestion of articular cartilage from the knee joints of 6-day-old newborn *ERN1* control and cKO mice. Briefly, after sacrificing the mice, we dislocated the femoral heads, femoral condyles and tibial plateau. The cartilage samples from one mouse were isolated under a microscope and then rinsed in PBS buffer. The cartilage samples were kept in a digestion buffer containing 0.25% collagenase II in DMEM for 12–16 h. After digestion, the cells were seeded in a 60 mm dish and allowed to grow in DMEM/F12 with 10% FBS and 100 units/ml streptomycin and penicillin for 3 days. Then, the medium was replaced with fresh medium.

### ACLT OA induction and intra-articular injection

OA was surgically induced in 10- to 12-week-old males by unilateral (right) anterior cruciate ligament transection (ACLT) surgery^3,4^. The ACL was transected to establish the ACLT model. The mice were sacrificed at the designated time points, and the knee joints were collected for analysis. The designated expression products were intra-articularly injected into the knee joints 4 weeks after surgery for 4 weeks. In the single treatment group, adenovirus expressing *ERN1* (Ad*ERN1*), XBP1s (Ad*XBP1s*) or control (AdGFP) was injected 1 time/week 4 times at 1 × 10^9^ PFU/time (<15 µl/joint). The combined treatment group, the Ad*ERN1*+si*PGRN* group, was injected with Ad*ERN1* two weeks after the surgery and then with *PGRN* siRNA (2.5 nm/10 μl/time, 1 time/week) 4 days later. The Ad*XBP1s*+rhPGRN group was injected with Ad*XBP1s* two weeks after the surgery and then with recombinant PGRN protein (4 μg/time, 1 time/week) 4 days later. The PGRN knockout (*GRN*^−/−^) mice were divided into three groups. The control group (AdGFP) was injected 1 time/week 4 times and 1 × 10^9^ PFU/time (<15 μl/joint). The Ad*ERN1* group was injected 1 time/week 4 times and 1 × 10^9^ PFU/time (<15 μl/joint). The Ad*ERN1*+rhPGRN group was injected with Ad*ERN1* 1 time/week 4 times after surgery and then with recombinant PGRN protein (4 μg/time, 1 time/week) simultaneously.

### CRISPR-Cas9 technique

The oligonucleotide sequences used for the generation of single guide RNAs (sgRNAs) are listed in Supplementary Table 1. The *ERN1*-KO and *PGRN*-KO cell lines were generated via the CRISPR‒Cas9 system. *ERN1*-KO and *PGRN*-KO monoclonal cell lines (KO1 and KO2) were generated using single-guide RNAs (Supplementary Table 1). Single-guide RNAs targeting human *ERN1* and *PGRN* were designed using E-CRISP (www.e-crisp.org/E-CRISP/designcrispr.html). Pairs of forward and reverse oligonucleotides for sgRNAs were annealed and then inserted into the plasmid vector LentiCRISPR v2, and 293T cells were transfected with sgRNA vector and two packaging plasmids (psPAX2 and pVSVG) using 100 mM PEI, pH 7.0 (Sigma #408727) diluted in ddH_2_O (pVSVG:psPAX2:lentiCRISPR=1:2:3). Supernatants were collected at 48 h post-transfection and passed through a 0.45 μm filter. Subsequently, the lentivirus supernatants were transduced into C28/I2 cells with 5 μg/ml polybrene. Forty-eight hours later, the cells were transferred and selected by 0.5 μg/ml puromycin for 3 days. Puromycin-resistant clones were sorted and confirmed by western blotting.

### Reverse transcription-PCR (RT‒PCR)

RT‒PCR and real-time PCR analyses were performed as previously described^16^. The relative mRNA expression was quantified using the comparative cycle threshold (CT) method, as previously described^16^. The full list of primers is listed in Supplementary Table 2.

### Immunofluorescence (IF) and immunohistochemistry (IHC)

IF and IHC analyses were performed to detect the expression of IRE1α, phosphorylated IRE1α, Col2, Aggrecan, MMP13, PGRN, XBP1s, and p-ERK1/2 in chondrocytes and cartilage as previously described^16^. The full list of primary antibodies is listed in Supplementary Table 3.

### Mass spectrometry analysis

C28/I2 cells were seeded in 10 cm dishes. When the cell density reached 90%, the medium was changed to serum-free medium, and the eukaryotic expression plasmid pcDNA3.1(−)-Myc-IRE1α was transfected into C28/I2 cells using Lipo2000. The serum-free medium was replaced with fresh DMEM containing 10% FBS after coincubation for 6–8 h. Then, after the culture was continued for 48 h, the original medium was removed, and the cells were washed three times with precooled PBS. Then, 400.0 μl of IP buffer was added, and the cells were lysed for 30 min on ice and shaken every 10 min during this time. The samples were centrifuged at 15,000 rpm and 4 °C for 15 min, the supernatant was collected, anti-Myc antibody was added, and the samples were incubated overnight on a 4 °C rotary mixer. Magnetic beads were then collected and washed five times with prechilled IP buffer for 5 min each. The beads were collected and analyzed for the label-free quantitative proteomics assay to assess the IRE1α-enriched proteins by Shanghai Bioprofile Technology Co., Ltd., Shanghai, China.

### Co-immunoprecipitation (Co-IP)

Cells were lysed with cold IP-lysis buffer (50 mM Tris-HCl, 150 mM NaCl, 2 mM EDTA, 0.5% NP40, pH 7.5) with protease inhibitors (1 mM PMSF) for 30 min as described previously^16^.

### RNA-seq library preparation and sequencing

RNA-seq library preparation and sequencing were performed in accordance with previous reports^7,18^.

### Luciferase assay

For generation of the pGL3-Col2-luc reporter plasmid, the promoter region of the human *Col2A1* gene was amplified by PCR using the genomic DNA of healthy adult volunteers’ peripheral venous blood as a template. The gene fragment was located between −1267 bp upstream and +285 bp downstream of the start of transcription, and PCR products were inserted into the pGL3 vector after digestion with the restriction endonucleases MluI and BglII. The primers are shown in Supplementary Table 1. For the luciferase assay, cells were cultured in 24-well plates and cotransfected with 0.5 μg of *Col2A1* gene-responsive luciferase reporter (pGL3-*Col2*) and *XBP1s* eukaryotic expression plasmid (pcDNA3.1-*XBP1s*) using PEI (Sigma) following the manufacturer’s instructions. The pRL-SV40 plasmid (Promega) was added as an internal control. Cells were harvested 48 h post-transfection and subjected to the Dual-Luciferase® Reporter Assay (Promega #E1910). Each assay was performed in triplicate and repeated three times.

### Chromatin immunoprecipitation (ChIP)

C28/I2 cells were cultured and then treated with formaldehyde (final 1%) at 37 °C for 10 min to crosslink the target protein and the corresponding genomic DNA. Glycine solution (final 0.125 M) was added to stop crosslinking. Cells were washed with cold PBS and lysed with ChIP lysis buffer (50 mM HEPES, 150 mM NaCl, 1 mM EDTA, 0.1% SDS, 0.1% sodium deoxycholate, 1% Triton X-100, pH 7.5). The lysate was sonicated to shear DNA to a length between 200 and 1000 bp. The sonicated supernatant was incubated with either antibody against XBP1s (Biolegend, USA) or control IgG overnight at 4 °C with rotation. For the collection of the DNA-XBP1s-antibody complex, Magrose Protein A/G (BEAVER) was added to the mixture and incubated for 1 h at 4 °C with rotation, and the DNA/Magrose Protein A/G complex was pelleted by a magnetic stand. After extensive washing of the pellet in a series of washing buffers, the pellet was dissolved in elution buffer (10 mM EDTA, 50 mM Tris, 1% SDS, pH 7.5), and the magnetic beads were removed. The supernatant was treated with NaCl (final 0.2 M) and heated to 65 °C for 4 h to reverse the protein‒DNA crosslinking. After treatment with EDTA and proteinase K, the supernatant was extracted with phenol/chloroform and precipitated with ethanol to recover the DNA. The DNA pellet was resuspended in a small amount of water for qPCR detection of the target gene. The full list of primers is listed in Supplementary Table 2.

### Western blot analysis

Western blot analysis was performed as described previously^16^. The full list of primary antibodies is listed in Supplementary Table 3.

### Scanning electron microscopy (SEM) and transmission electron microscopy (TEM)

Femoral heads were isolated from 12-month-old control mice and *ERN1* cKO mice. Femoral heads were treated with 0.1% (w/v) trypsin (Sigma #T7409) and 0.1% (w/v) hyaluronidase (Sigma #H3506) at 37 °C for 24 h each to remove proteoglycans, fixed with Karnovsky’s fixative at room temperature for 3 h, and sequentially dehydrated in graded water–ethanol and tert-butyl alcohol. The freeze dryer was precooled to approximately −50 °C, and the samples with tert-butanol were placed in and defrosted for 15–30 min. The samples were vacuumed until the tert-butanol was completely sublimated, approximately 3–8 h. The samples were then coated with platinum and imaged using a scanning electron microscope (SU8010, Hitachi).

Then, costal cartilage was isolated from 12-month-old control mice and *ERN1* cKO mice. All samples were fixed with 2% glutaraldehyde solution and decalcified with 15% (w/v) EDTA at pH 7.4 for 14 days. After dehydration with an alcohol gradient series, each sample was doubly replaced with propylene oxide, soaked with epoxy resin, and embedded in an oven at 60 °C for 48 h. The specimens were then sectioned into ultrathin slices, dyed with citric acid lead, and examined under a transmission electron microscope (JEM-1400Plus, JEOL).

### Sulfated glycosaminoglycan assay (sGAG)

Mouse knee joint cartilage samples were collected at 4 weeks after ACLT surgery and lyophilized. Explants (20–50 mg) were digested with papain (20 mg/ml) in 0.2 M sodium phosphate buffer (Na_2_HPO_4_–NaH_2_PO_4_, pH 6.4), 8 mg/ml sodium acetate, 4 mg/ml EDTA and 0.8 mg/ml L-cysteine-HCl at 65 °C for 18 h. The tubes were centrifuged at 10,000×*g* for 10 min. The supernatant was decanted for use with a Blyscan sGAG assay (Blyscan, Biocolor, Ltd., Carrickfergus, UK) with a chondroitin sulfate standard and normalized against the explants’ wet weight. sGAG absorbance readings were carried out for each cartilage sample in duplicate in 96-well plates at 656 nm. The S-glycosaminoglycan concentrations were obtained from the standard curve.

### Statistical analysis

All statistical analyses were analyzed using GraphPad Prism software (version 7.0; GraphPad, Inc., La Jolla, CA, USA). Student’s *t-*test was used to establish statistical significance between the two groups. One-way or two-way analysis of variance (ANOVA) was performed. Experimental data are expressed as the mean ± standard error of the mean (S.E.M.). A *P* value <0.05 was considered statistically significant.

## Results

### The expression profile of IRE1α in OA patients and mice with surgically induced OA

To identify the potential role of IRE1α/XBP1s in OA, we isolated primary cartilage tissues from human osteoarthritic and control articular cartilage, and the protein expression levels of IRE1α, p-IRE1α and XBP1s in the articular cartilage of patients with OA were significantly higher than those in the non-OA cartilage (Supplementary Fig. 1). Furthermore, the expression of IRE1α and phosphorylated IRE1α in the articular cartilage of the mice in the ACLT model group was also notably higher and more widely distributed than that in the sham group (Supplementary Fig. 2). The data indicate that the articular cartilage microenvironment of OA exists in a state of ER stress. The expression profile of IRE1α/XBP1s is associated with the occurrence and development of OA, and thus, it was further investigated.

### Specific deficiency of *ERN1* in chondrocytes generates an OA-like phenotype in aging mice

**Fig. 1:**
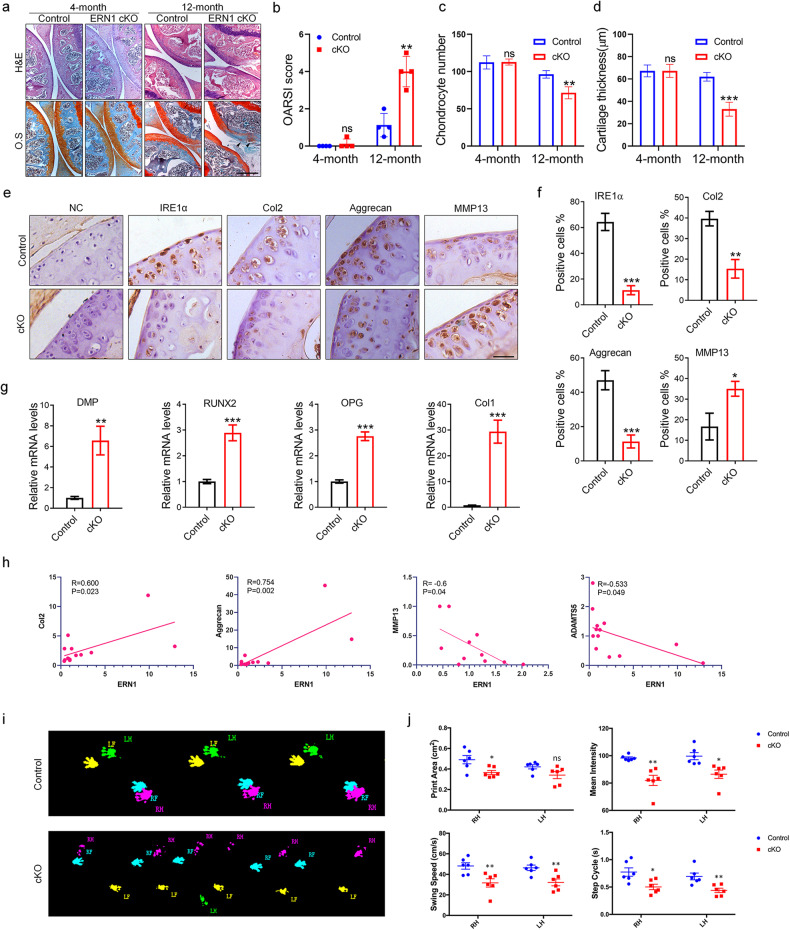
*ERN1* cKO mice spontaneously developed an osteoarthritis-like phenotype with aging. Knee joint samples were collected from 4- and 12-month-old *ERN1* cKO mice and control mice for HE staining and safranin fast green staining. Significant proteoglycan loss in *ERN1* cKO mice is indicated (black arrow), scale bar: 350.0 μm (**a**), and the OARSI score (**b**), chondrocyte number (**c**) and articular cartilage layer thickness (**d**) in the tibia were determined. *N* = 4 for each group. Immunohistochemistry was used to observe the expression and distribution of IRE1α, Col2, Aggrecan and MMP13 in the knee joint pathological sections of *ERN1* cKO mice and control mice (*n* = 4), scale bar: 200.0 μm (**e**) and quantitative data of positive cells (**f**). Expression of osteogenesis-related markers in primary chondrocytes from *ERN1* cKO mice and primary chondrocytes from wild-type mice as controls (**g**). The mRNA expression levels of *Col2* and *Aggrecan* were positively correlated with *ERN1* expression levels, and the mRNA expression levels of *MMP13* and *ADAMTS5* were negatively correlated with *ERN1* expression levels in the cartilage of mice (**h**). The Catwalk system was used to analyze the gait of 12-month-old *ERN1* cKO mice and control mice (**i**) and to quantify the print area, mean intensity, swing speed, and step cycle of mice (**j**). The gait data are expressed as the mean ± SEM. ns not significant; *P < 0.05, **P < 0.01, and ***P < 0.001.

We then generated mice with cartilage-specific defects in *ERN1* (*ERN1*^flox/flox^*Col*_*2*_Cre^+^, *ERN1* cKO mice) (Supplementary Fig. 3a, b) and observed spontaneous OA development and symptoms in aging *ERN1* cKO mice (Supplementary Video). The hind limb joints of 4-month-old and 12-month-old mice were collected. As shown in Fig. 1a–d, in the 12-month-old mouse group, proteoglycans, chondrocyte number and cartilage thickness in the articular cartilage of the *ERN1* cKO mice were significantly lower than those of the control littermates, and the Osteoarthritis Research Society International (OARSI) score indicated more severe OA symptoms in the *ERN1* cKO mice than in the control mice, although there was no significant difference in the 4-month-old mice. In addition, IHC results showed that compared with those of control mice, articular cartilage anabolic markers, including *Col2* and *Aggrecan*, were decreased in the *ERN1* cKO mice, whereas the catabolic marker *MMP13* was increased (Fig. 1e, f).

Simultaneously, the expression of osteogenesis-related markers, such as *DMP*, *RUNX2*, *OPG*, and *Col1*, was upregulated in primary chondrocytes isolated from the cartilage of the *ERN1* cKO mice compared to that in control primary chondrocytes from the cartilage of the wild-type mice (Fig. 1g). Pearson correlation analysis showed that the mRNA level of *ERN1* was positively associated with the mRNA levels of *Col2* and *Aggrecan* and negatively related to the levels of *MMP13* and *ADAMTS5* in the mouse cartilage tissues (Fig. 1h). The gait analysis of 12-month-old *ERN1* cKO mice and control mice further indicated that the print area, swing speed, mean intensity, step cycle, run average speed, stand, max contact and max intensity of the *ERN1* cKO mice were clearly reduced, whereas the run duration, stride length and swing of the *ERN1* cKO mice were obviously increased compared with those of the control mice. These findings indicated that *ERN1* cKO mice may suffer from OA-related pain (Fig. 1i, j, Supplementary Fig. 4). These data confirm that *ERN1* plays a protective role in OA progression.

### *ERN1* deficiency causes aggravated OA development in a surgically induced arthritis model

**Fig. 2:**
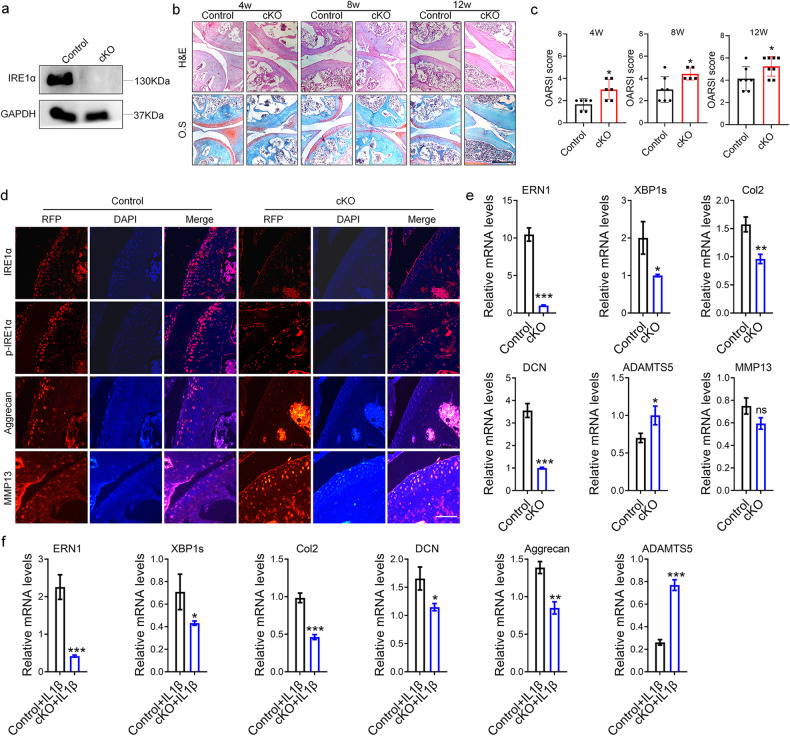
*ERN1* cKO mice have more severe cartilage damage in the ACLT-induced arthritis model. The expression of IRE1α in primary chondrocytes of the *ERN1 cKO* mice and control mice was detected by western blotting (**a**). In the 4th, 8th, and 12th weeks after ACLT modeling, joint samples from the *ERN1* cKO group mice and the control group mice were collected for HE staining and safranin fast green staining (*n* = 6), scale bar: 350.0 μm (**b**), and the OARSI score (**c**) was determined. **d** Immunofluorescence staining was performed on the mouse samples of each group 12 weeks after ACLT modeling to observe the expression of IRE1α, p-IRE1α, Aggrecan, and MMP13 in the articular cartilage area, scale bar: 100.0 μm. The expression levels of *ERN1*, *XBP1s*, *Col2*, *DCN*, *ADAMTS5* and *MMP13* in primary chondrocytes of the *ERN1* cKO mice and control mice were detected by RT‒qPCR (**e**). Furthermore, RT‒qPCR was used to determine the expression levels of *ERN1*, *XBP1s*, *Col2*, *DCN*, *Aggrecan*, and *ADAMTS5* in the two groups of cells under IL1β-induced inflammation (**f**). ns not significant; *P < 0.05, **P < 0.01, and ***P < 0.001.

We first verified that the expression of IRE1α in primary chondrocytes of *ERN1* cKO mice was significantly reduced by Western blotting (Fig. 2a). Then, ACLT-induced OA models were established in control and *ERN1* cKO mice, and samples were collected at predetermined time points (4, 8 and 12 weeks). The OARSI score indicated that the *ERN1* cKO mice displayed more severe OA symptoms than the control mice after ACLT surgery (Fig. 2b, c). The results showed that with the extension of time after surgery, more severe knee cartilage damage was observed in the *ERN1* cKO mice than in the control mice, as indicated by the destruction of cartilage integrity and loss of proteoglycans. Furthermore, immunofluorescence staining was performed on the knee joint tissue samples of the mice at 12 weeks (Fig. 2d). The levels of total and phosphorylated IRE1α in the articular cartilage of mice in the *ERN1* cKO group were significantly lower than those in the control group. Moreover, the expression of the cartilage anabolic marker aggrecan in the articular cartilage of the *ERN1* cKO mice was significantly lower than that of the control group, while the expression of the cartilage catabolic marker MMP13 was significantly higher than that of the control group. Compared with that of the primary chondrocytes of control mice, after successful *ERN1* knockout, the mRNA level of *XBP1s* decreased significantly. In addition, the mRNA level of the cartilage anabolic markers *Col2* and *DCN* in the primary chondrocytes of *ERN1* cKO mice were significantly reduced, while the expression of the catabolic marker *ADAMTS5* was significantly increased (Fig. 2e). Moreover, under IL-1β-induced inflammatory conditions, the cartilage anabolic markers were more obviously reduced, and the catabolic marker was clearly enhanced in the primary chondrocytes of the *ERN1* cKO mice (Fig. 2f). Subsequently, stable cell lines with specific *ERN1* knockout (*ERN1* KO1 and *ERN1* KO2) were constructed and screened by CRISPR‒Cas9 in human C28/I2 cells (Supplementary Fig. 5a). The RT‒qPCR data showed that, compared with that of the control group, the cartilage anabolic marker *DCN* was significantly reduced after *ERN1* was knocked out, while the cartilage catabolic marker *MMP13* was significantly increased (Supplementary Fig. 5b). In addition, under inflammatory conditions, the decrease in the cartilage anabolic markers *Col2* and *DCN* and the increase in the decomposition marker *MMP13* were more obvious in the *ERN1* null chondrocytes (Supplementary Fig. 5c).

Furthermore, we found that overexpression of *ERN1* (Ad*ERN1*), with increased phosphorylation of IRE1α, induced significant increases in cartilage anabolic markers, such as *Col2* and *aggrecan*, while the cartilage catabolic marker *MMP13* was significantly decreased. This phenomenon was notable in the inflammatory microenvironment induced by IL-1β, which manifested as an obvious enhancement in cartilage anabolic markers and a decrease in cartilage catabolic markers after IL-1β induction (Supplementary Fig. 6a–d). In summary, *ERN1* can protect cartilage by promoting cartilage anabolism, inhibiting catabolism, and relieving the progression of OA.

### IRE1α binds to PGRN, which is required for the chondroprotective effect of IRE1α

**Fig. 3:**
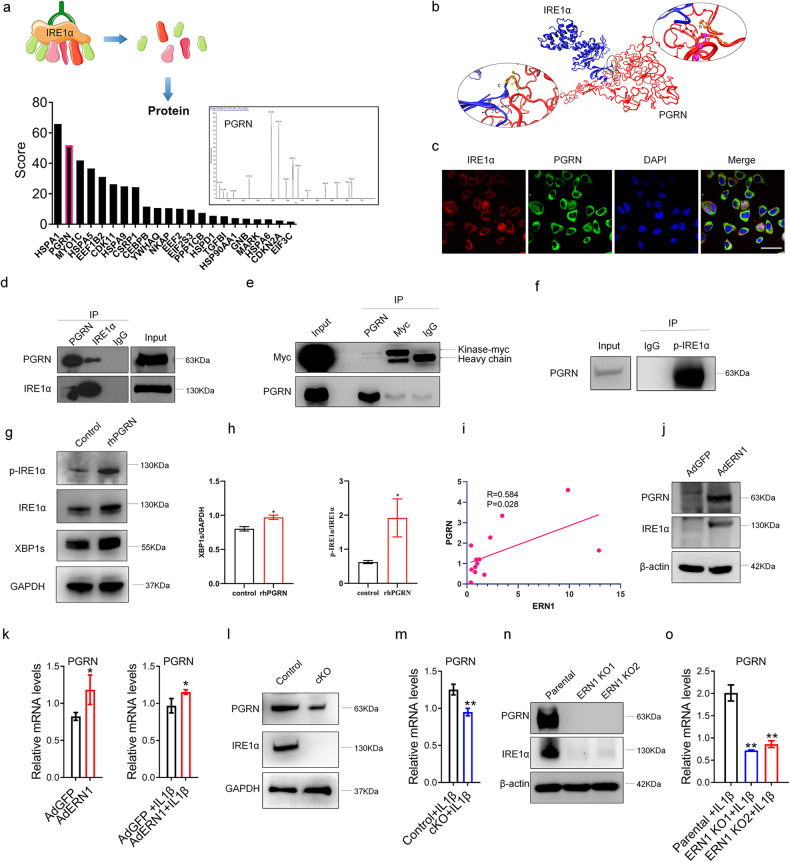
IRE1α binds to PGRN and regulates its expression. PGRN can bind to IRE1α through screening of nonlabeled protein quantitative technology (**a**). The interaction of IRE1α with the two subunits of PGRN was predicted using the visual molecular dynamics (VMD) technique (**b**). The intracellular distribution of IRE1α and PGRN was observed by fluorescence colocalization, scale bar: 100.0 μm (**c**). Co-IP technology verified that PGRN and IRE1α can bind to each other (**d**). Co-IP technology further showed that the IRE1α kinase domain is the binding region of PGRN (**e**). Co-IP technology verified that p-IRE1α can bind to PGRN (**f**). The expression of p-IRE1α, IRE1α and XBP1s in C28/I2 cells with or without rhPGRN treatment was detected by western blotting and quantified (**g**, **h**). The mRNA levels of *PGRN* were positively correlated with *ERN1* expression levels in the mouse cartilage (*n* = 13) (**i**). The expression of PGRN after AdERN1 was overexpressed in C28/I2 cells and was detected by western blotting (**j**). RT‒qPCR was used to detect the expression of *PGRN* after Ad*ERN1* was overexpressed in C28/I2 cells under normal or inflammatory conditions (**k**). The expression of PGRN in primary chondrocytes of the *ERN1* cKO mice and control mice was detected by western blotting (**l**). RT‒qPCR was used to detect the expression of *PGRN* in primary chondrocytes of the *ERN1* cKO mice and control mice under inflammatory conditions (**m**). The expression of PGRN in *ERN1* KO C28/I2 cells was detected by western blotting (**n**). RT‒qPCR was used to detect the expression of *PGRN* in *ERN1* KO C28/I2 cells under inflammatory conditions (**o**). ns not significant; *P < 0.05, **P < 0.01.

To explore the mechanism of IRE1α in the pathogenesis of OA, we performed a proteomic analysis to detect and identify IRE1α-binding proteins. Mass spectrometry data revealed that PGRN is a protein that is highly enriched for binding to IRE1α (Fig. 3a). We then used a visual molecular dynamics (VMD) technique to predict that IRE1α interacts with the two subunits of PGRN (Fig. 3b). Subsequently, fluorescence colocalization revealed that IRE1α and PGRN were colocalized in the cytoplasm (Fig. 3c). The co-immunoprecipitation data were consistent with the VMD results, which confirmed that IRE1α and PGRN could bind to each other (Fig. 3d). A previous report showed that IRE1α contains a kinase (524 aa-827 aa) and an RNase (828 aa-977 aa) domain. We further revealed that the kinase domain of IRE1α binds to PGRN (Fig. 3e) and that the RNase domain cannot bind to PGRN (Supplementary Fig. 7). Then, we detected the interaction between p-IRE1α and PGRN. The results showed that p-IRE1α can bind to PGRN (Fig. 3f), and PGRN promotes the expression of p-IRE1α, IRE1α and XBP1s (Fig. 3g, h). Furthermore, Pearson correlation analysis showed that the mRNA level of *ERN1* was positively associated with the mRNA level of *PGRN* in mouse cartilage tissues (Fig. 3i). Moreover, overexpression of IRE1α upregulated the mRNA and protein expression of PGRN, and the same trend was observed under IL-1β-induced inflammatory conditions (Fig. 3j, k). The protein level of PGRN was significantly decreased in the *ERN1*-defect primary chondrocytes and *ERN1*-deficient stable cell lines compared with the controls (Fig. 3l). Importantly, the mRNA expression of *PGRN* in the *ERN1*-deficient chondrocytes was significantly downregulated under IL-1β-induced inflammatory conditions (Fig. 3m–o). The above data reveal that IRE1α and PGRN can bind to each other, and their expression profiles affect each other and change due to the influence of inflammatory factors. Additionally, the expression of PGRN in osteoarthritic cartilage tissue was significantly increased compared with that in normal cartilage tissue, consistent with the changes in the expression profiles of IRE1α, p-IRE1α, and XBP1s in the articular cartilage of OA patients, suggesting that PGRN and IRE1α are the key proteins involved in the progression of OA (Supplementary Figs. 1 and 8).

**Fig. 4:**
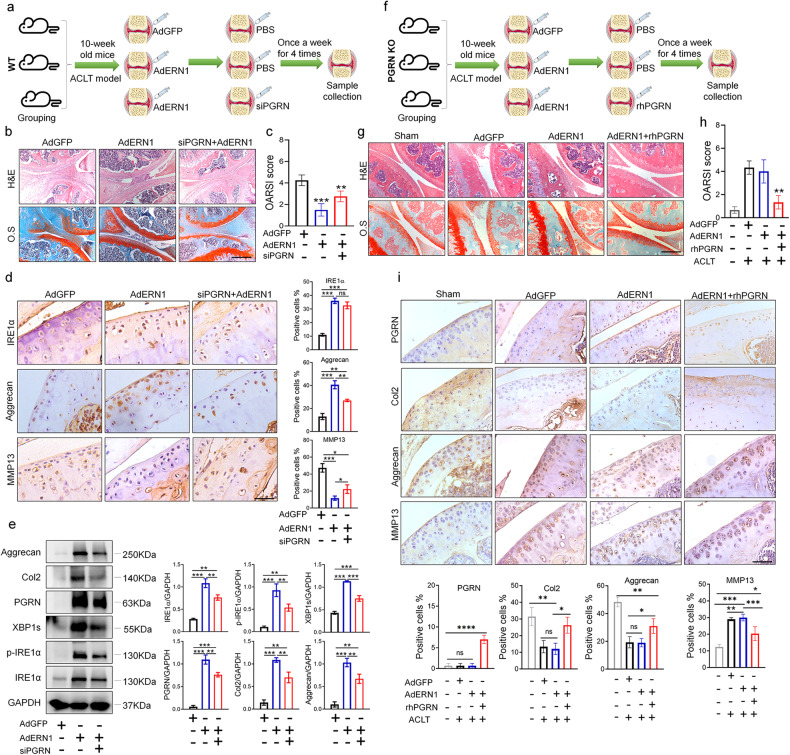
Knockdown and loss of PGRN in ACLT-induced arthritis model mice can inhibit the chondroprotective effect of Ad*ERN1*. **a** Schematic diagram of the treatment plan for the ACLT-induced arthritis model of C57 wild-type mice. Mouse joint samples from the Ad*GFP*, Ad*ERN1*, and si*PGRN*+Ad*ERN1* treatment groups were collected for H&E and safranin fast green staining (*n* = 4); scale bar: 350.0 μm (**b**), and the OARSI score was determined (**c**). The expression levels of IRE1α, Aggrecan, and MMP13 in the articular cartilage tissue of each group of C57 wild-type mice were detected by immunohistochemistry (*n* = 4), scale bar: 50.0 μm, and the proportion of positive cells was quantified (**d**). The protein expression levels of IRE1α, p-IRE1α, XBP1s, PGRN, Col2, and Aggrecan in the knee joint cartilage samples of each group of C57BL/6J mice were detected by Western blotting, and quantitative analysis was performed by ImageJ software (**e**). ns not significant; *P < 0.05, **P < 0.01, and ***P < 0.001. **f** Schematic diagram of the treatment plan for the ACLT-induced arthritis model of *GRN*^-/-^ KO mice. Joint samples were collected from *GRN*^-/-^ KO mice and the control sham group, and intra-articular injections of AdGFP, Ad*ERN1*, and Ad*ERN1*+rhPGRN were performed for H&E and safranin fast green staining (*n* = 4). Scale bar: 350.0 μm (**g**). OARSI score (**h**). The expression levels of PGRN, Col2, Aggrecan, and MMP13 in the articular cartilage tissue of each group of *GRN*^-/-^ KO mice were detected by immunohistochemistry (*n* = 4), scale bar: 200.0 μm, and the proportion of positive cells/area was quantified (**i**). ns not significant; *P < 0.05, **P < 0.01, and ***P < 0.001.

To further determine whether and how PGRN participates in the mechanism by which Ad*ERN1* protects articular cartilage, we studied the treatment effect of Ad*ERN1* in the ACLT-induced OA model in C57 wild-type mice and *GRN*^-/-^ knockout (PGRN KO) mice. Ad*ERN1* and Ad*ERN1*+si*PGRN* were injected into the knee joint cavity of WT C57 mice, while Ad*ERN1* and Ad*ERN1*+rhPGRN were injected into the knee joint cavity of *GRN*^-/-^ KO mice after ACLT (Fig. 4a, f). The results showed that local delivery of Ad*ERN1* protected articular cartilage, while knockdown of PGRN inhibited the protective effect of Ad*ERN1* on cartilage to a certain extent in the WT ACLT mouse model (Fig. 4b, c), and Ad*ERN1* had no obvious chondroprotective effect in the *GRN*^-/-^ KO mouse model. The OARSI score showed that there was no significant difference between the arthritis score of the Ad*ERN1* group and that of the Ad*GFP* group in the *GRN*^-/-^ KO mouse ACLT model, indicating that PGRN deficiency inhibited the chondroprotective effect of Ad*ERN1* on cartilage. The local delivery of Ad*ERN1*+rhPGRN restored the chondroprotective effect on articular cartilage in the *GRN*^-/-^ KO mouse ACLT model (Fig. 4g, h).

IHC showed that in the WT ACLT mouse model, the number of aggrecan-positive cells on the cartilage surface of the Ad*ERN1* group was significantly higher than that of the control group. After PGRN knockdown, the number of aggrecan-positive cells decreased. In contrast, the number of MMP13-positive cells in the Ad*ERN1* group was significantly lower than that in the control group, while after knockdown of PGRN, the number of MMP13-positive cells increased (Fig. 4d). Moreover, in the *GRN*^-/-^ KO mouse ACLT model, Ad*ERN1* did not upregulate the expression of aggrecan and Col2 or downregulate that of MMP13, whereas rhPGRN recovered the above effects of Ad*ERN1* in the Ad*ERN1*+rhPGRN group (Fig. 4i). Furthermore, Western blot analysis showed that the protein levels of aggrecan, Col2, PGRN, XBP1s, and IRE1α/p-IRE1α were increased in the Ad*ERN1* group compared to the control group, whereas the expression of the above proteins was reduced after PGRN knockdown (Fig. 4e). Collectively, PGRN is involved in the protective effect of Ad*ERN1* on cartilage in mice, and the chondroprotective effect of IRE1α is dependent on PGRN.

### IRE1α accelerates XBP1s splicing and PGRN promotes the chondroprotective role of Ad*XBP1s* through ERK1/2 signaling

**Fig. 5:**
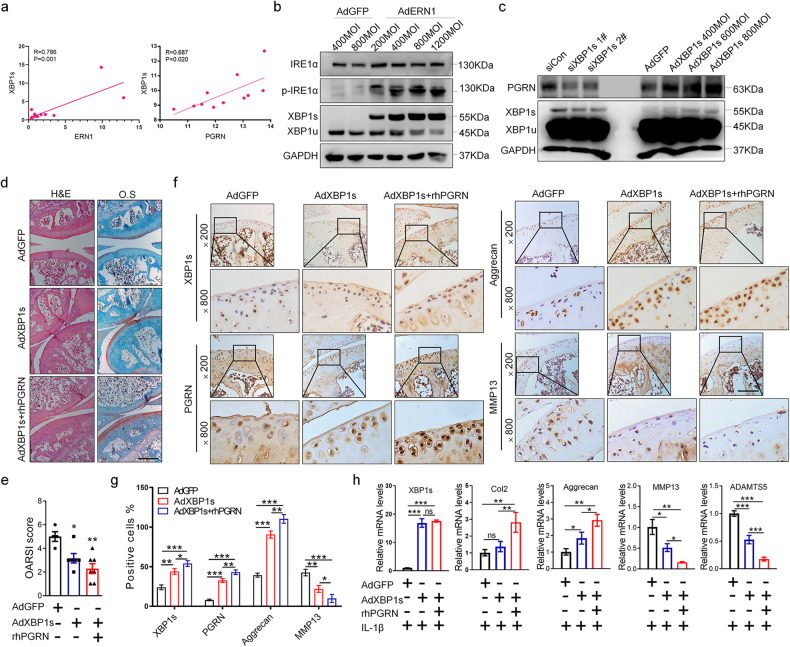
Overexpression of Ad*XBP1s* protects cartilage, and PGRN promotes its chondroprotective effect. The mRNA expression levels of *ERN1* and *PGRN* were positively correlated with XBP1s expression levels in the cartilage of mice (*n* = 11) (**a**). The expression levels of XBP1u, XBP1s, IRE1α, and p-IRE1α were detected by Western blots after adding different concentrations of Ad*ERN1* to C28/I2 cells (**b**). The expression levels of PGRN and XBP1s/u after knocking down *XBP1s* or overexpressing Ad*XBP1s* in C28/I2 cells were detected by Western blots (**c**). Knee joint samples of C57 wild-type mice in the Ad*GFP*, Ad*XBP1s*, and Ad*XBP1s*+rhPGRN treatment groups were stained with H&E and safranin (*n* = 6), scale bar: 350.0 μm (**d**), and the OARSI score was determined (**e**). Immunohistochemical staining was performed on the knee joint samples of each group of mice to observe the expression and distribution of XBP1s, PGRN, Aggrecan and MMP13 in cartilage tissue (*n* = 6), scale bar: 200.0 μm (**f**), and quantify the proportion of positive cells (**g**). RT‒qPCR was used to detect the mRNA levels of *XBP1s*, *Col2*, *Aggrecan*, *MMP13*, and *ADAMTS5* after Ad*XBP1s* was infected in OA patient cartilage tissue explants under IL-1β-induced inflammatory conditions with or without 200 ng/ml rhPGRN (**h**). ns not significant; *P < 0.05, **P < 0.01, and ***P < 0.001.

**Fig. 6:**
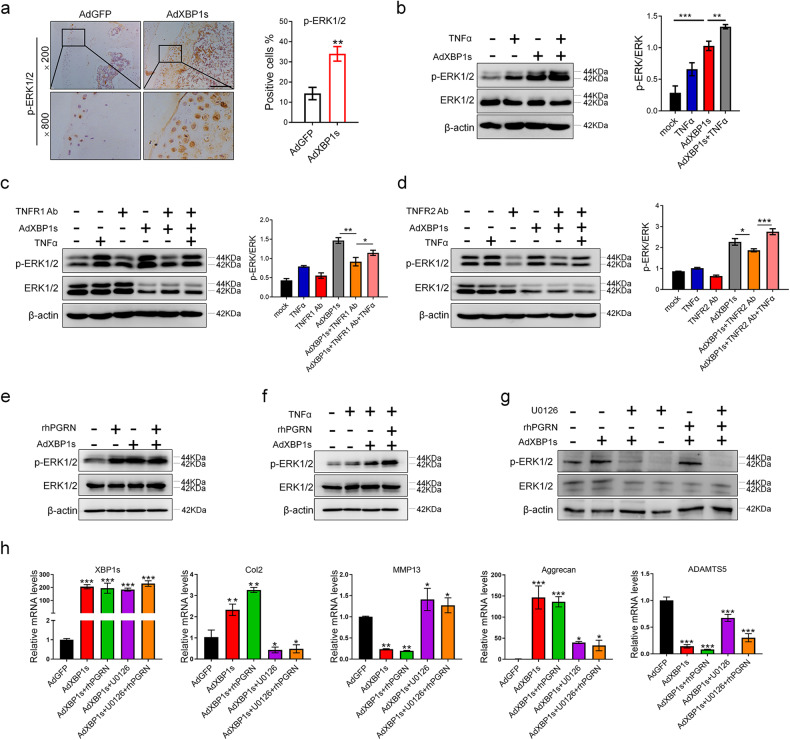
Overexpression of Ad*XBP1s* and PGRN affects cartilage metabolism through ERK1/2 signaling. The expression of p-ERK1/2 was detected by immunohistochemistry in knee joint cartilage of C57BL/6J mice with ACLT-induced arthritis (*n* = 6), scale bar: 200.0 μm (**a**). C28/I2 cells were treated with or without 10 ng/ml TNFα in the presence or absence of Ad*XBP1s* infection for 30 min. The expression levels of ERK1/2 and p-ERK1/2 were detected by Western blots, and quantitative analysis was performed (**b**). C28/I2 cells were starved with MEM-α medium for 6 h after infection with or without Ad*XBP1s* and then treated with 10 ng/ml TNFα for 30 min after incubation with 5 μg/ml TNFR1 antibody (TNFR1 Ab) (**c**) or 3 μg/ml TNFR2 antibody (TNFR2 Ab) (**d**) for 18 h. The cell lysates were collected, the levels of ERK1/2 and p-ERK1/2 were detected by Western blotting, and quantitative analysis was performed. C28/I2 cells were treated with or without 200 ng/ml rhPGRN for 1 h and infected with Ad*XBP1s* for 24 h (**e**). C28/I2 cells were treated with or without 200 ng/ml rhPGRN for 1 h with or without Ad*XBP1s* infection for 24 h and then treated with 10 ng/ml TNFα for 15 min (**f**). The expression levels of ERK1/2 and p-ERK1/2 were detected by Western blotting. C28/I2 cells were treated with 30 μM U0126 regardless of whether Ad*XBP1s* was infected for 24 h with or without 200 ng/ml rhPGRN for 1 h. The cell lysates were collected, the levels of ERK1/2 and p-ERK1/2 were detected by Western blotting (**g**), and the mRNA expression levels of *XBP1s*, *Col2*, *Aggrecan*, *MMP13* and *ADAMTS5* were detected by RT‒qPCR (**h**). *P < 0.05, **P < 0.01, and ***P < 0.001.

Next, Pearson correlation analysis showed that the mRNA level of *XBP1s* was positively associated with the mRNA levels of *ERN1* and *PGRN* in mouse cartilage tissues (Fig. 5a). Western blot analysis showed that overexpression of Ad*ERN1* promoted the production of phosphorylated IRE1α and XBP1s in a dose-dependent manner. Overexpression of Ad*XBP1s* also increased the protein level of PGRN, whereas si*XBP1s* inhibited the expression of PGRN (Fig. 5b, c). Notably, PGRN modulates the expression profile of IRE1α and XBP1s with positive feedback. We next explored whether and how PGRN affects the function of XBP1s. As shown in Fig. 5d and e, Ad*XBP1s* and Ad*XBP1s*+rhPGRN were injected into the knee joint cavity of WT C57 mice after ACLT. The results showed that compared with that of the Ad*GFP* control group, the arthritis score of the Ad*XBP1s* group was significantly lower, and the arthritis score of the Ad*XBP1s*+rhPGRN group was significantly lower than that of the Ad*XBP1s* group. IHC results also showed that XBP1s upregulates the expression of PGRN, the number of aggrecan-positive cells on the cartilage surface of the Ad*XBP1s* group was higher than that of the Ad*GFP* control group, and the value of the Ad*XBP1s*+rhPGRN group was significantly higher than that of the Ad*XBP1s* group; the number of MMP13-positive cells on the cartilage surface of the Ad*XBP1s* group was reduced compared with that of the Ad*GFP* control group, and the value of the Ad*XBP1s*+rhPGRN group was significantly decreased compared with that of the Ad*XBP1s* group (Fig. 5f, g). These data indicate that overexpression of Ad*XBP1s* can protect articular cartilage and that rhPGRN can promote the chondroprotective effect of Ad*XBP1s* in vivo. Moreover, overexpression of XBP1s increased the mRNA levels of *Aggrecan* and *Col2* and decreased the mRNA levels of *ADAMTS5* and *MMP13* in cartilage tissue explants from OA patients. Then, rhPGRN promoted the upregulation of *Aggrecan* and *Col2* by AdXBP1s and improved the downregulation of *ADAMTS5* and *MMP13* by AdXBP1s in the AdXBP1s+rhPGRN group under IL-1β-induced inflammatory conditions (Fig. 5h). As reported in the literature, PGRN activation of extracellular signal-regulated kinases (ERK1/2) is involved in the regulation of numerous processes, and ERK1/2 is known to protect chondrocytes from OA^19–21^. We then found that overexpression of XBP1s can activate the phosphorylation of the ERK1/2 signaling pathway in cartilage and chondrocytes with or without TNFα-induced inflammatory conditions (Fig. 6a, b). In addition, XBP1s can activate ERK1/2 signaling phosphorylation when the TNF-α effect is blocked or partially blocked by the TNFR1/2 antibody (Fig. 6c, d). Moreover, PGRN increased the phosphorylation of ERK1/2 activated by XBP1s, and this promoting effect was more obvious under TNFα-induced inflammatory conditions (Fig. 6e, f). Importantly, ERK1/2 phosphorylation activated by AdXBP1s and AdXBP1s+rhPGRN was blocked after U0126 treatment in chondrocytes. Furthermore, after inhibition of ERK1/2 phosphorylation in chondrocytes by U0126, the upregulation of Aggrecan and Col2 by AdXBP1s and AdXBP1s+rhPGRN and the downregulation of ADAMTS5 and MMP13 by AdXBP1s and AdXBP1s+rhPGRN were blocked. The protective articular cartilage roles of AdXBP1s and AdXBP1s+rhPGRN were dependent on ERK1/2 signaling (Fig. 6g, h). Collectively, XBP1s and XBP1s+PGRN exerted chondroprotective effects by activating ERK1/2 signaling.

### *ERN1* regulates collagen homeostasis by controlling collagen II expression through *XBP1s*

**Fig. 7:**
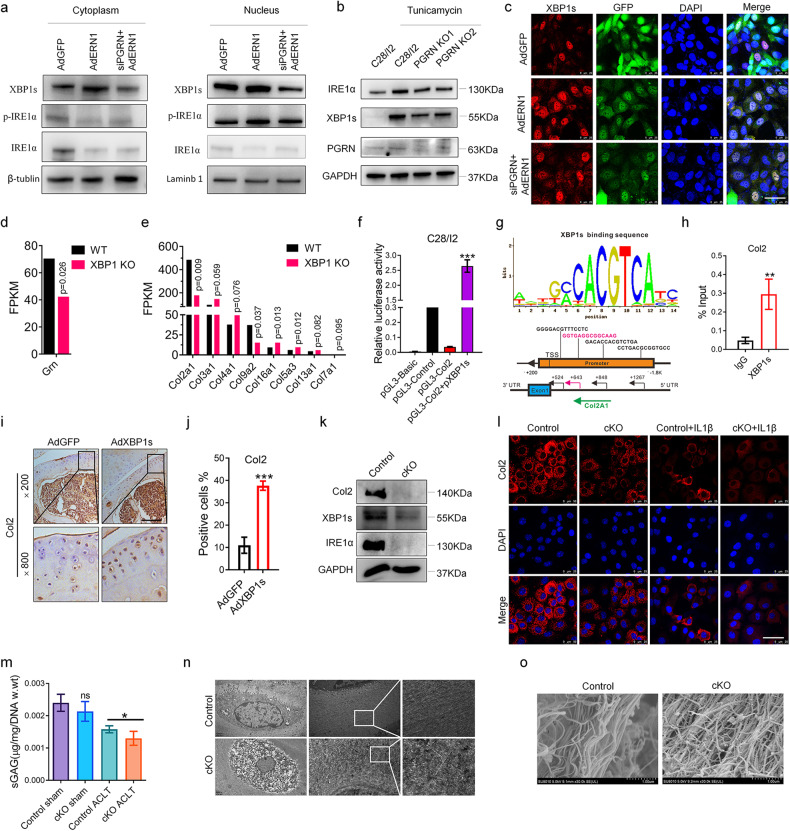
XBP1s nuclear translocation is dependent on PGRN, and XBP1s maintains collagen homeostasis by upregulating Col2 expression. The effects of Ad*ERN1* and Ad*ERN1*+si*PGRN* with TM (20 ng/µl) on the distribution of XBP1s, p-IRE1α, and IRE1α in the nucleus and cytoplasm were detected by Western blotting (**a**). The expression levels of IRE1α, XBP1s and XBP1u in the *PGRN* KO cell line were detected by Western blotting (**b**). The effects of Ad*GFP*, Ad*ERN1*, and Ad*ERN1*+si*PGRN* with TM (20 ng/µl) on the intracellular distribution of XBP1s were observed by immunofluorescence; scale bar: 100.0 μm (**c**). The differential expression of mRNAs between *XBP1* KO cartilage and normal cartilage was identified through whole transcriptome RNA-seq (**d**, **e**). The regulation of the expression of *Col2* by XBP1s was detected by the dual-fluorescence enzyme reporter gene (**f**). The potential XBP1s binding site for the *Col2A1* gene sequence was predicted using Jaspar (http://jaspar.genereg.net/) (**g**). Chromatin immunoprecipitation-qPCR (ChIP‒qPCR). With the *XBP1s* DNA binding sequence as a substrate, four pairs of primers were designed for qPCR; the input and IgG groups were used as controls (**h**). The expression and distribution of Col2 in mouse knee cartilage after overexpression of XBP1s were detected by immunohistochemistry (*n* = 6), scale bar: 200.0 μm (**i**), and the proportion of positive cells was quantified (**j**). The expression of Col2 in primary chondrocytes of the *ERN1* cKO mice and control mice was detected by Western blotting (**k**). The expression and distribution of Col2 in primary chondrocytes of the *ERN1* cKO mice and control mice under normal and inflammatory conditions were observed by immunofluorescence, scale bar: 100.0 μm (**l**). Sulfated glycosaminoglycan (sGAG) synthesis assay. Mouse femoral head cartilage was isolated from the control and *ERN1* cKO mice after ACLT surgery for 4 weeks and then detected by GAG synthesis assays and standardized by DNA (**m**). The ultrastructure of femoral head cartilage in the *ERN1* cKO mice and control mice was observed by transmission electron microscopy, ×5000 (**n**) and scanning electron microscopy, ×30000, scale bar: 1.0 μm (**o**). ns not significant; *P < 0.05, **P < 0.01, and ***P < 0.001.

Previous studies have confirmed that XBP1s, a transcription factor with multiple regulatory functions, enters the nucleus to regulate the transcription and expression of related genes after splicing by phosphorylated IRE1α^22,23^. As shown in Fig. 7a, overexpression of Ad*ERN1* increased the phosphorylation level of IRE1α and the spliced protein XBP1s in the nucleus under ER stress but had no significant effect on the protein level of XBP1s or phosphorylated IRE1α in the cytoplasm. Importantly, after the knockdown of *PGRN* with siRNA, overexpression of Ad*ERN1* did not enhance the phosphorylation of IRE1α or the spliced protein level of XBP1s in the nucleus. In addition, in *PGRN* knockout chondrocytes constructed with the CRISPR‒Cas9 technique, the protein levels of IRE1α and XBP1u were obviously reduced compared to those of wild-type chondrocytes, whereas XBP1s, resulting from XBP1u splicing, was only clearly detectable under ER stress (Fig. 7b). During IL-1β-induced ER stress, the transcription factor XBP1s, spliced by phosphorylated IRE1α, undergoes nuclear translocation and regulates the transcription of related genes in the nucleus. As shown by cellular immunofluorescence, Ad*ERN1* increased the expression of XBP1s in the nucleus after IL-1β treatment. However, si*PGRN* suppressed the nuclear translocation of XBP1s (Fig. 7c). Moreover, the nuclear translocation of XBP1s increased after rhPGRN treatment (Supplementary Fig. 9). Thus, the splicing and nuclear translocation of XBP1s are dependent on PGRN.

As reported by Cameron et al., cartilage-specific deficiency of *XBP1* in mice causes chondrodysplasia characterized by reduced chondrocyte proliferation and delayed cartilage maturation and mineralization^24^. We constructed six mRNA libraries between *XBP1* KO cartilage and normal cartilage and then identified the different mRNAs through whole transcriptome RNA-seq^18^. We compared some differentially expressed collagen-related proteins, including Col2a1, Col9a2, and Col16a1. The FPKM (fragments per kilobase of transcript sequence per million base pairs sequenced) expression of *Col2a1* was the most significantly different in the *XBP1*-deficient chondrocytes, and its expression was significantly reduced compared with that of the control (*p* = 0.009). In addition, the FPKM expression of *PGRN* was significantly decreased compared with that of the control (*p* = 0.026) (Fig. 7d, e). We next predicted the binding site of the transcription factor XBP1s to the *Col2a1* promoter, and chromatin immunoprecipitation (ChIP) analysis confirmed that XBP1s could bind to the *Col2a1* (−1267 to +285) promoter regions. Furthermore, overexpression of the transcription factor XBP1s significantly upregulated the transcriptional activity of the *Col2a1* reporter gene (pGL3-*Col2*) (Fig. 7f–h). Similarly, IHC data showed that the expression of Col2 was significantly higher than that in the control group in the articular cartilage of mice after XBP1s was overexpressed by injection of Ad*XBP1s* into the articular cavity (Fig. 7i, j). Additionally, in *ERN1*-null chondrocytes, *ERN1* deficiency led to reduced XBP1s expression in chondrocytes, and the expression of Col2 was also significantly suppressed (Fig. 7k). Immunofluorescence analysis of primary chondrocytes revealed that the expression of Col2 in the *ERN1 cKO* group was lower than that in the control group with or without IL-1β treatment, and Col2 expression in the *ERN1* cKO group decreased more significantly than that in the control group (Fig. 7l). This finding further shows that IRE1α/*ERN1* promotes the expression of *Col2* through the XBP1s signaling axis to protect cartilage. Moreover, the sGAG assay showed that the level of sGAG in the *ERN1* cKO group was lower than that in the control group, and there was no significant difference between the control and cKO sham groups; however, after ACLT surgery, the level of sGAG in the *ERN1* cKO group was reduced more significantly than that in the control group. Proteoglycans constituting the extracellular matrix (ECM) and articular cartilage were significantly reduced and degraded in the ACLT-induced model of *ERN1*-deficient mice compared to control mice (Fig. 7m). We next observed the ultrastructure of collagen in cartilage and chondrocytes using TEM and SEM. TEM data revealed that the organelles of chondrocytes were destroyed when *ERN1* was deficient, including nuclear condensation and marginalized nuclear chromatin, increased vacuolization in the cytoplasm, disrupted ER, and disordered collagen arrangement around chondrocytes (Fig. 7n). The SEM results were consistent with the TEM data, showing that the cartilage collagen fibers were discontinuous, had a rough surface, and had a disordered arrangement in the cartilage of the *ERN1-*deficient mice compared with that in the control mice (Fig. 7o). Collectively, we found that in *ERN1*-deficient chondrocytes, collagen homeostasis is imbalanced and that the ultrastructure is abnormal. We further show that *ERN1* maintains collagen homeostasis by regulating *Col2* transcription and expression through the transcription factor XBP1s.

## Discussion

Osteoarthritis is primarily characterized by progressive breakdown, loss of extracellular matrix proteins and collagen fibers, and articular cartilage damage caused by various factors, including aging and injury^25,26^. ER stress has been confirmed to be related to the occurrence and development of many diseases, including OA^27,28^, RA^16,29^, and other inflammatory diseases^30,31^. IRE1α is a classic sensor of the UPR, which is involved in the folding of secreted proteins in the ER, including collagens II, IX, and X, proteoglycans and other proteins that make up the ECM. According to the heterogeneity and complexity of OA, the expression profiles of several UPR genes, including IRE1α and XBP1s/u, presented obvious differences with different OA patients and disease courses. This finding is also confirmed by the high expression of IRE1α and its phosphorylation we observed (Supplementary Fig. 1) and the differential expression profiles reported in the literature^16,32^. The highly conserved UPR molecules present a complex and variable expression profile with the course of OA, suggesting the importance of individualized and precise treatment of OA.

We observed and found that older *ERN1*-specific knockout mice were more prone to spontaneously developing OA symptoms, such as severe proteoglycan loss, more obvious cartilage damage, joint pain, and abnormal gait, than control mice. In addition, degenerative biomarkers, including MMP13, were significantly elevated, whereas levels of the anabolic markers aggrecan and Col2 were critically decreased in the cartilage of the *ERN1* cKO mice compared with that of the control group with aging (Fig. 1a–f, h)^33^. Type II collagen-positive (Col2^+^) progenitors are the major source of endochondral ossification, and they are major stem cells that control skeletal development and vascular formation^34^. Col2a1-Cre was shown to target chondrocytes and osteoblastic lineage cells, including osteocytes, osteoblasts, and their precursors/progenitors, in long bones^35^. Herein, we observed that the expression of some osteocyte marker genes, including DMP, OPG and Col1, was upregulated in the cartilage tissue of *ERN1* CKO mice (Fig. 1g). These findings suggested that Col2Cre-specific chondrocyte lineage defects in the *ERN1* gene may be involved in cartilage degeneration and damage by affecting endochondral ossification. In the *ERN1* cKO ACLT mouse model, more severe loss of proteoglycan in safranin O staining, a significantly higher arthritis score, elevated expression of MMP13 and *ADAMTS5*, and reduced expression of aggrecan and *Col2* were observed (Fig. 2). Combined with these data, the loss of IRE1α in chondrocytes may accelerate OA aging and degeneration. *ERN1* plays a protective role in articular cartilage.

Through proteomics analysis, we identified that PGRN binds to IRE1α and participates in the regulation of IRE1α on chondrocyte metabolism. PGRN has been implicated in a variety of physiological processes and pathological states and has multiple functions^36–39^. The unique domains or motifs of PGRN determine the diversity of proteins that bind to it and thus the functional diversity of PGRN. Previous reports have shown that most PGRN-related proteins can be divided into the following three categories^40^: cell membrane receptors, ECM molecules, and intracellular chaperones and lysosomal hydrolases^39,41–49^. In this study, we detected and identified a novel PGRN-binding protein, IRE1α. PGRN, as an intracellular chaperone, binds to the kinase domain of IRE1α and assists in the phosphorylation of IRE1α to promote the splicing of XBP1u to generate XBP1s during ER stress (Fig. 3). Intriguingly, IRE1α achieves its chondroprotective effect by combining with PGRN and promoting XBP1s splicing. This chondroprotective effect of IRE1α is dependent on PGRN and is achieved through upregulation of ERK1/2 signaling (Figs. 4–6). Moreover, the expression profiles of PGRN, IRE1α and XBP1s are positively correlated and can influence each other. They can form a positive feedback regulatory loop. Intra-articular injection of rhPGRN protein has been reported to attenuate OA-like phenotypes and relieve OA progression in mice. This therapeutic effect of PGRN is mediated through interactions with the TNF-α and β-catenin signaling pathways^50^. Herein, PGRN is associated with IRE1α/XBP1s, the most conserved signaling pathway in ER stress, and plays a protective role in cartilage via positive feedback regulation with IRE1α/XBP1s during the process of OA. PGRN assists IRE1α in realizing the splicing and nuclear translocation of XBP1s. Activated XBP1s then translocates to the nucleus, where it functions as a multifunctional transcription factor. Cameron et al. reported that cartilage-specific XBP1-deficient mice exhibit chondrodysplasia, and the UPR pathway presents genetic redundancy in cartilage pathology, thereby mediating the XBP1-independent UPR signaling pathway^10,24^. Although the XBP1-independent UPR signaling pathway may compensate for some of the defects in XBP1s function due to *ERN1* deficiency, the role and mechanism of how *ERN1* deficiency and XBP1-dependent UPR signaling affect other downstream molecules to regulate cartilage metabolism in *ERN1-*deficient mice is unclear. Of note, we demonstrate that PGRN serves as a master regulator of cartilage metabolism in OA, compensating for some of the functions lost due to *ERN1* deficiency by promoting and upregulating the biological effects of XBP1s. Moreover, the protective articular cartilage roles of XBP1s and XBP1s+PGRN were dependent on ERK1/2 signaling (Fig. 6), which is involved in the regulation of cell proliferation and survival. These results again confirm our previous findings^15,51^. Huang et al. also showed that deficiency of IRE1α in chondrocytes downregulates the prosurvival factors XBP1s and Bcl-2, which promotes the apoptosis of chondrocytes^17^. In addition to survival and apoptosis, collagen homeostasis imbalance and loss are major features of cartilage senescence, and aging is the main cause of OA. Intriguingly, we found that XBP1s can maintain collagen homeostasis by regulating the expression of type II collagen, thus participating in the protection against cartilage degeneration in OA (Fig. 7).

**Fig. 8:**
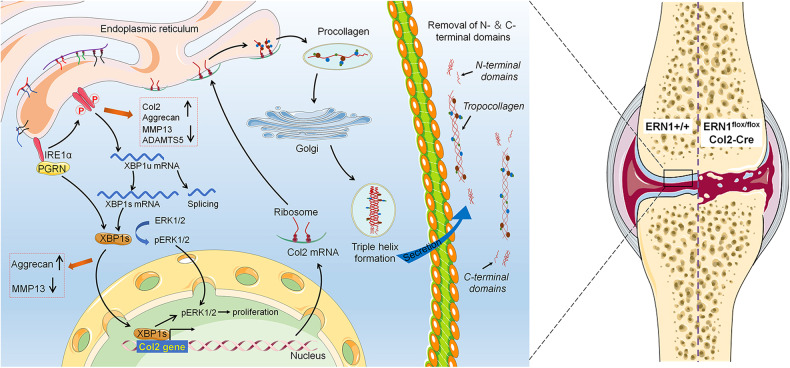
Schematic diagram of the regulation of IRE1α in the occurrence and development of osteoarthritis. PGRN binds to IRE1α and promotes IRE1α phosphorylation; later, p-IRE1α splices XBP1u to generate XBP1s, which undergoes nuclear translocation, subsequently upregulating the expression of Col2 and maintaining collagen homeostasis, achieving a chondroprotective effect by activating ERK1/2 signaling. *ERN1*/IRE1α plays a protective role in cartilage by regulating PGRN-dependent XBP1 splicing and collagen homeostasis.

As summarized in Fig. 8, IRE1α binds to PGRN and splices XBP1u to generate XBP1s, which undergoes nuclear translocation, subsequently upregulating the expression of Col2 to maintain collagen homeostasis and achieve a chondroprotective effect. Furthermore, the chondroprotective effect of IRE1α relies on PGRN. Moreover, XBP1s exerts a chondroprotective role by activating ERK1/2 signaling, and PGRN promotes this effect. Enhancement of XBP1s function by PGRN may play a compensatory role in the absence of *ERN1*/XBP1s function. *ERN1* deficiency accelerates cartilage degeneration in OA by reducing PGRN expression and XBP1 splicing, which in turn decreases the expression of type II collagen, resulting in collagen structural abnormalities and an imbalance in collagen homeostasis. The exploration of the mechanisms underlying OA development and pathogenesis is important for the clinical individualized precision treatment of OA. This study provides the key role of IRE1α in the progression of OA and presents new insights into the role of IRE1α in the treatment of degenerative joint diseases.

### Supplementary information

Supplementary Material

The phenotypic traits of ERN1 control and ERN1 CKO mice
