# Supplementary material for: IRE1α protects against osteoarthritis by regulating progranulin-dependent XBP1 splicing and collagen homeostasis
Source: Exp Mol Med. 2023 Nov 1;55(11):2376–89. doi: 10.1038/s12276-023-01106-w (PMC10689778; doi:10.1038/s12276-023-01106-w)
Supplement: Supplementary file 1 — Supplementary Material [file 12276_2023_1106_MOESM1_ESM.pdf]

IRE1α protects against osteoarthritis by regulating progranulin-dependent XBP1 splicing and collagen homeostasis

Running title: IRE1α protects against osteoarthritis

Li Liang[1#], Fengmei Zhang[1,2#], Naibo Feng[1#], Biao Kuang[3], Mengtian Fan[1], Cheng Chen[4], Yiming Pan[1], Pengfei Zhou[5], Nana Geng[1], Xingyue Li[1], Menglin Xian[1], Lin Deng[1], Xiaoli Li[1], Liang Kuang[6], Fengtao Luo[6], Qiaoyan Tan[6], Yangli Xie[6], Fengjin Guo[1,*]

[1] Laboratory of Developmental Biology, Department of Cell Biology and Genetics, School of Basic Medical Sciences, Chongqing Medical University, Chongqing 400016, China.

[2] Laboratory Animal Center, Chongqing Medical University, Chongqing 400016, PR China

[3] Department of Orthopedics, the 2nd Affiliated Hospital of Chongqing Medical University, Chongqing, 400016, China

[4] Department of Orthopedics, the 1st Affiliated Hospital of Chongqing Medical University, Chongqing, 400016, China

[5] Chongqing Key Laboratory of Oral Diseases and Biomedical Sciences, College of Stomatology, Chongqing Medical University, Chongqing 400016, China

[6] Department of Wound Repair and Rehabilitation Medicine, Center of Bone Metabolism and Repair (CBMR), State Key Laboratory of Trauma and Chemical Poisoning, Research Institute of Surgery, Daping Hospital, Army Medical University, Chongqing 400042, China

[#] These authors contributed equally to this work.

Corresponding Author

Fengjin Guo, Laboratory of Developmental Biology, Department of Cell Biology and Genetics, School of Basic Medical Sciences, Chongqing Medical University, Chongqing 400016, China.

E-mail: guo.fengjin@cqmu.edu.cn; guo.fengjin@gmail.com.

SUPPLEMENTARY FIGURES

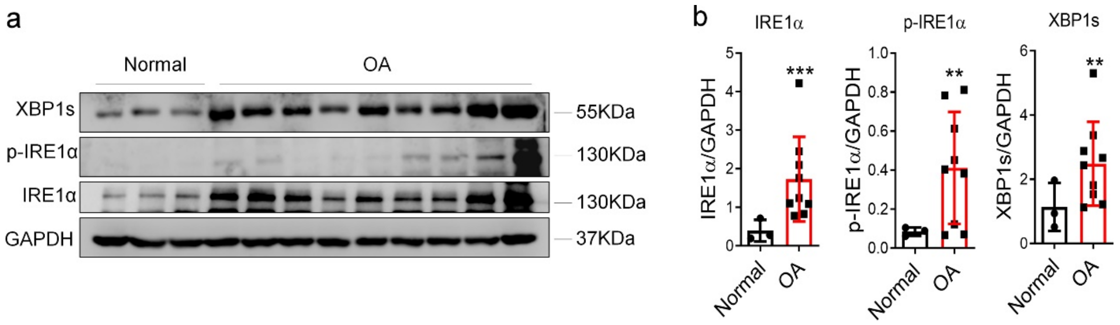

Supplementary Fig. 1. The expression of IRE1α, p-IRE1α, and XBP1s were measured in non-OA (Normal) and OA patients knee joints cartilage by Western blot (a) and quantitative analysis by Image J software (b). $**P<0.01$, and $***P<0.001$.

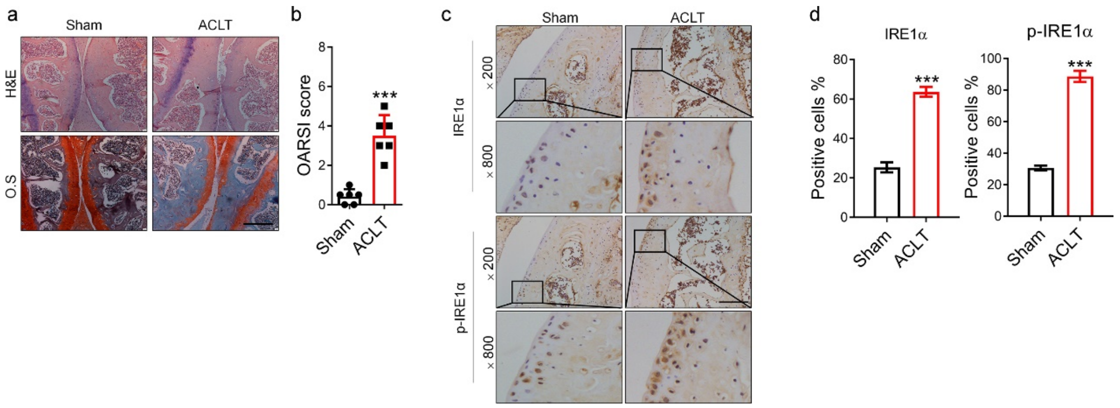

Supplementary Fig. 2. C57BL/6 J mice for 4 weeks after ACLT surgery were collected for HE staining and safranin-fast green staining (n=6), scale bar: 350.0 μm (a), and OARSI score (b) was performed. The expression of IRE1α and p-IRE1α were detected by immunohistochemistry in knee joint sections of C57BL/6 J mice for 4 weeks after ACLT surgery (n=6), scale bar: 200.0 μm (c) and the proportion of positive cells was quantified (d). ***P*<0.01, and ****P*<0.001.

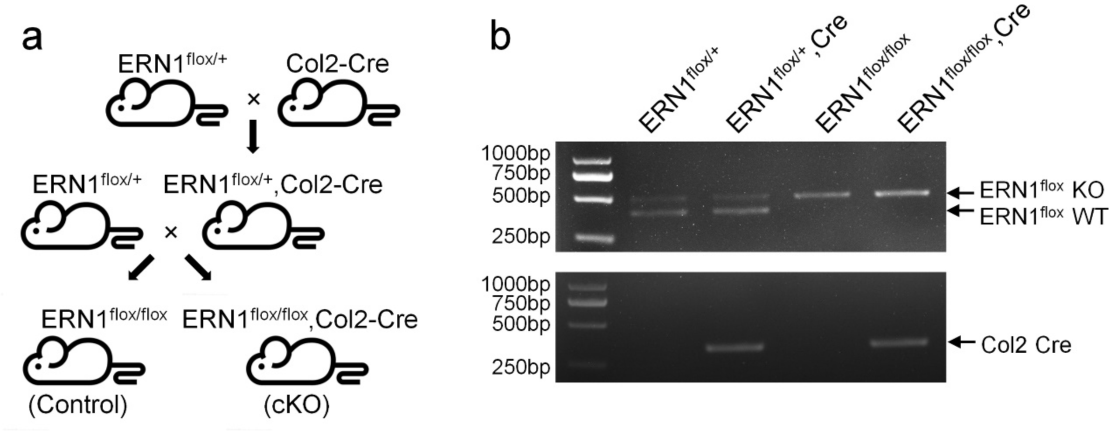

Supplementary Fig. 3. Schematic diagram of ERN1 cKO mouse construction (a) and genotype identification results (b).

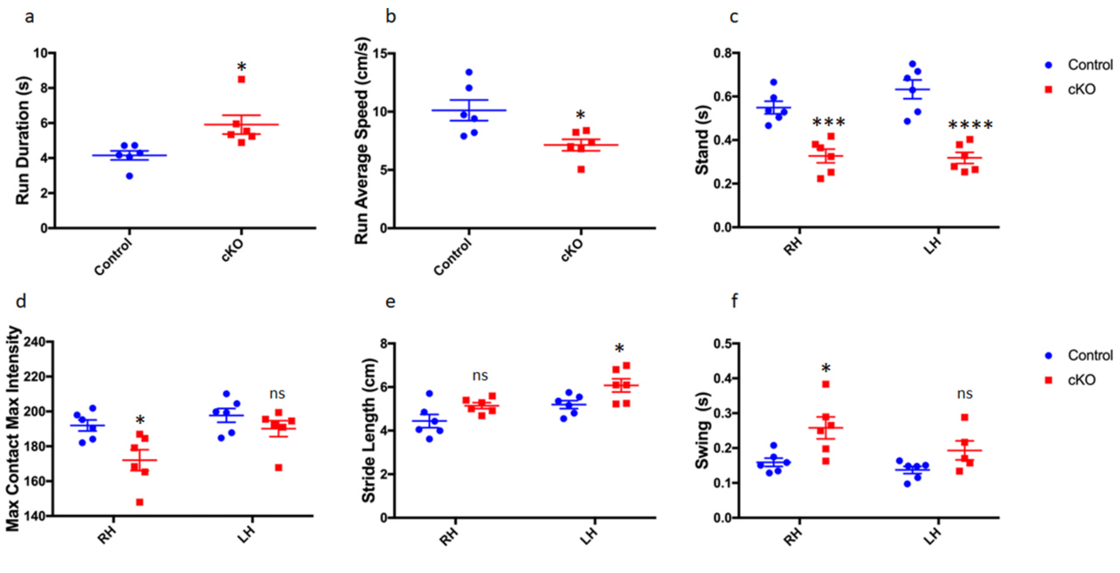

Supplementary Fig. 4. The Catwalk system was used to analyze the gait of 12-month-old *ERN1* cKO mice and control mice and to quantify the run duration (a), run average speed (b), stand (c), max contact max intensity (d), stride length (e) and swing (f) of mice. ns, not significant; *P<0.05, ***P<0.001, and ****P<0.0001 .

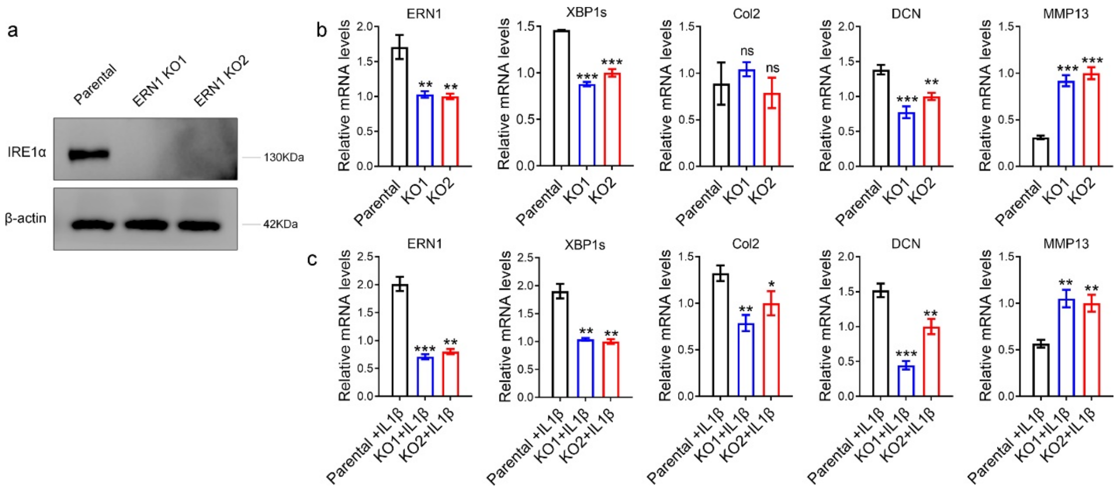

Supplementary Fig. 5. Assay of chondrocyte metabolic marker gene in *ERN1* knockout C28/I2 cells. Western blot confirmation of ERN1 knockout in C28/I2 cells (a), the mRNA levels of *ERN1, XBP1s, Col2, DCN, and MMP13* in C28/I2 knockout *ERN1* cells were detected by RT-qPCR(b). Under inflammatory conditions, the mRNA levels of *ERN1*, *XBP1s*, *Col2*, *DCN* and *MMP13* in C28/I2 knockout *ERN1* cells were detected by RT-qPCR (c). ns, not significant; **P*<0.05, ***P*<0.01, and ****P*<0.001.

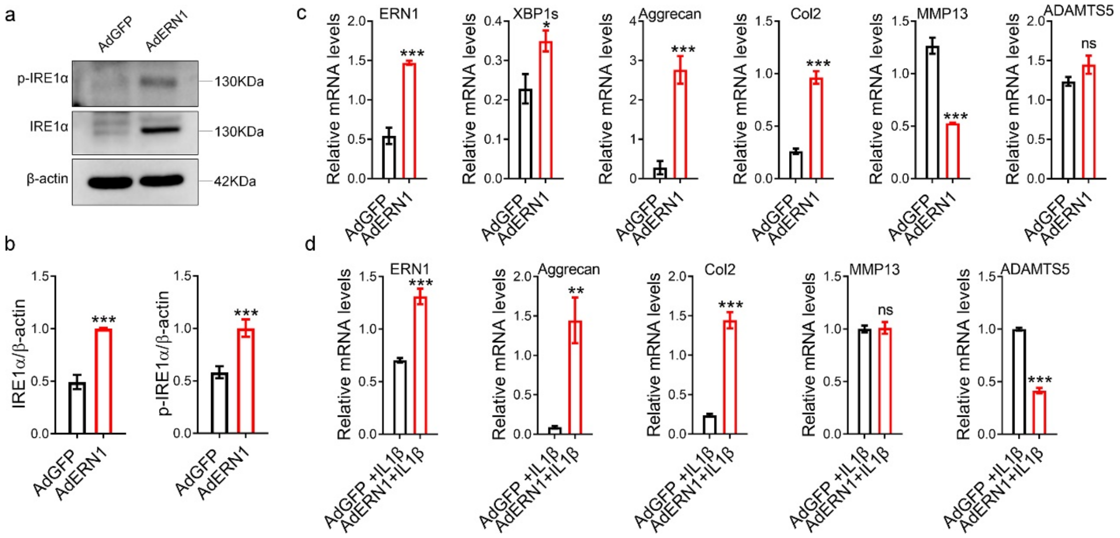

Supplementary Fig. 6. Overexpression of Ad*ERN1* influences on the catabolism and anabolism in C28I2 cells. The expression of IRE1α and its phosphorylation level after overexpression of Ad*ERN1* in C28/I2 cells were detected by Western blot (a), and quantitative analysis was performed by Image J software (b). The mRNA levels of *ERN1*, *XBP1s*, *Aggrecan*, *Col2*, *MMP13*, and *ADAMTS5* after AdERN1 overexpression in C28/I2 cells were detected by RT-qPCR (c). Under inflammatory conditions, the mRNA expression levels of *ERN1*, *Aggrecan*, *ADAMTS5*, *MMP13*, and *Col2* were detected by RT-qPCR after overexpression of Ad*ERN1* in C28/I2 cells (d). ns, not significant; **P*<0.05, ***P*<0.01, and ****P*<0.001.

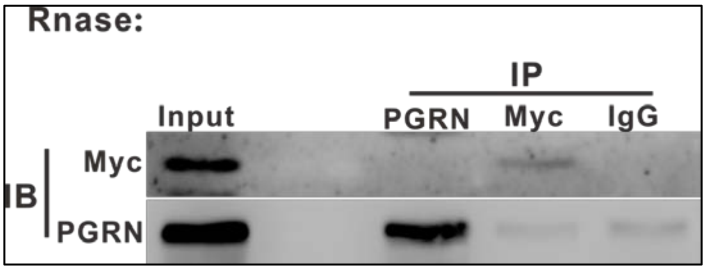

Supplementary Fig. 7. The CO-IP results for the RNase (828aa-977aa) domain of IRE1α and PGRN.

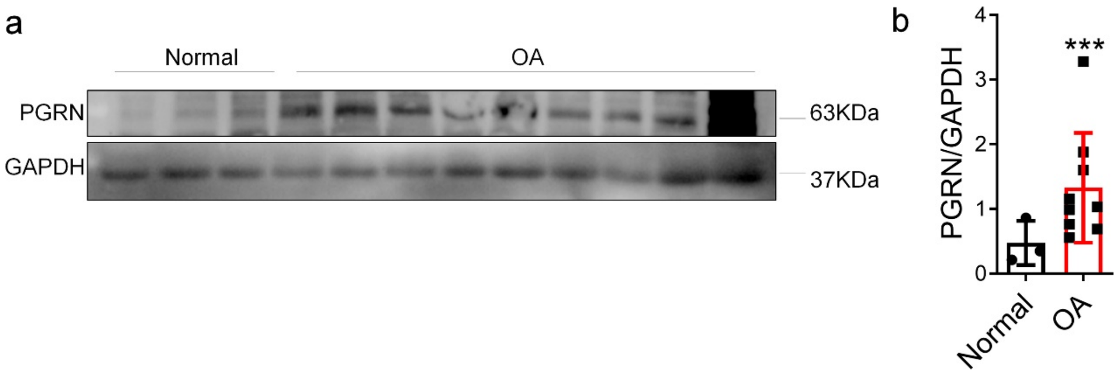

Supplementary Fig. 8. The expression of PGRN were measured in non-OA (Normal) and OA patients knee joints cartilage by Western blot (a) and quantitative analysis by Image J software (b). ****P*<0.001.

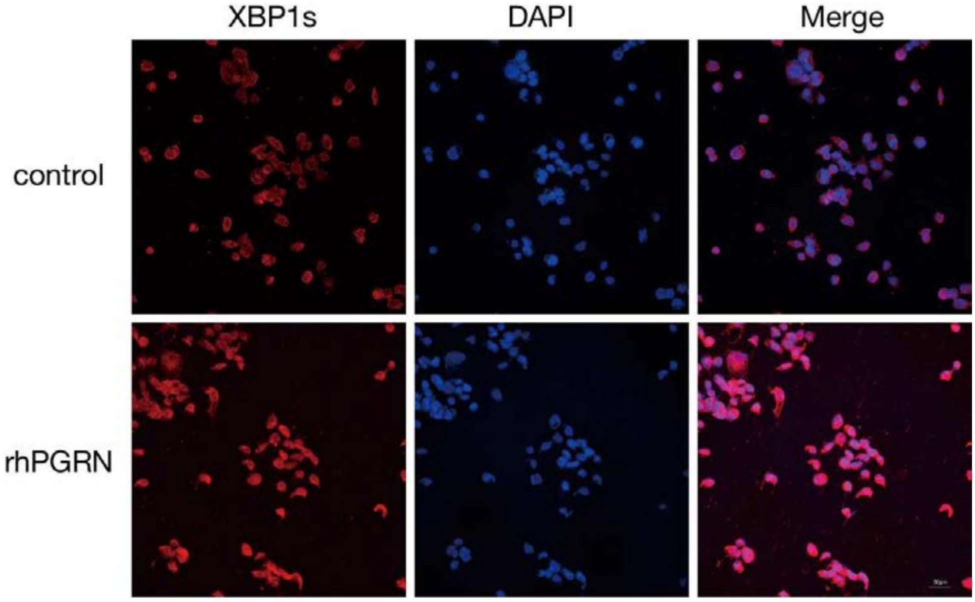

Supplementary Fig. 9. The effect of rhPGRN on the intracellular distribution of XBP1s was observed by immunofluorescence, scale bar: 100.0 μm.

Supplementary Table 1. The following primer sequences (5'-3') are used:

**Cas9 primers**	
sgERN1 1F	CACCGACATCCCGAGACACGGTGGT
sgERN1 1R	AAACACCACCGTGTCTCGGGATGTC
sgERN1 2F	CACCGCTTAAGCATGGAGTCCACGG
sgERN1 2R	AAACCCGTGGACTCCATGCTTAAGC
sgPGRN 1F	CACCGCTGATGGCACGCTCACCTC
sgPGRN 1R	AAACGAGGTGAGCGTGCCATCAGC
sgPGRN 2F	CACCGCAGCTGTAGCTGGCTCCTC
sgPGRN 2R	AAACGAGGAGCCAGCTACAGCTGC
**siRNA sequences**	
siPGRN(mouse) sense（5'-3'）	CCCGCUCCAAAUUGGAGAUAUtt
siPGRN(mouse) antisense（5'-3'）	AUAUCUCCAAUUUGGAGCGGGtt
modifications used in siRNAs including 2'-OMe,5’-PS, 5’-Chol, improve stability	
**clone primers**	
pGL3-Col2 promoter-1267→+285 (F)	CCGAGCTCTT ACGCGT ATTATGAATCGCAGGGAC
pGL3-Col2 promoter-1267→+285 (R)	TACTTAGATCGCAGATCTAGGCTGGAGTTCCAAGT
**CHIP qPCR primers**	
Col2 1F	GTGGGACACTCATCTCGG
Col2 1R	CAGTGGGGTAGGGTCAA
Col2 2F	TTTTCAGTTCAGTGGCTCC
Col2 2R	CGAAGGTCAGTGGGGTAG
Col2 3F	GGCATTGACCCTACCC
Col2 3R	GCAGTTGTTGATGAAGCA

Col2 4F	CAGTTTCTTGGGTATGGTAT
Col2 4R	GCAACGGGAATTGACA
ps: Predicted promoter binding site:NC_000012.12:c48008212-48006012	

Supplementary Table 2. The following primer sequences are used:

Primer	Sequence
**Genotyping primers**	
ERN1 Flox (F)	ACCCAAGAGAAGGAAGCCAGAGA
ERN1 Flox (R)	CCAGGGTCGAGACAAACAACAAG
Col2-Cre (F)	GAGGGTCCAGCCCGAGCTACTT
Col2-Cre (R)	GCATCGACCGGTAATGCAGGC
**qPCR primers**	
GAPDH (M-F)	AGGTCGGTGTGAACGGATTTG
GAPDH (M-R)	TGTAGACCATGTAGTTGAGGTCA
ERN1 (M-F)	ACACCGACCACCGTATCTCA
ERN1 (M-R)	CTCAGGATAATGGTAGCCATGTC
XBP1s (M-F)	GCTGAGTCCGCAGCAGGTG
XBP1s (M-R)	GGTCCAACTTGTCCAGAATGCC
PGRN (M-F)	TGAAGAGTGATACACCTTGTGATGA
PGRN (M-R)	TTCTGACAGTACCCCTGAGCC
Aggrecan (M-F)	CCTGCTACTTCATCGACCCC
Aggrecan (M-R)	AGATGCTGTTGACTCGAACCT
COL2 (M-F)	CAGGATGCCCGAAAATTAGGG
COL2 (M-R)	ACCACGATCACCTCTGGGT
DCN (M-F)	TCTTGGGCTGGACCATTTGAA
DCN (M-R)	CATCGGTAGGGGCACATAGA
ADAMTS (M-F)	GGAGCGAGGCCATTTACAAC
ADAMTS (M-R)	CGTAGACAAGGTAGCCCACTTT
MMP13 (M-F)	CTTCTTCTTGTTGAGCTGGACTC
MMP13 (M-R)	CTGTGGAGGTCACTGTAGACT
DMP (M-F)	CATTCTCCTTGTGTTCCTTTGG
DMP(M-R)	TCAGTATTGTGGTATCTGGCAACT
OPG (M-F)	ACGGACAGCTGGCACACCAG

OPG(M-R)	CTCACACACTCGGTTGTGGG
RUNX2 (M-F)	CCGTGGCCTTCAAGGTTGT
RUNX2(M-R)	TTCATAACAGCGGAGGCATTT
Col1 (M-F)	GCGAAGGCAACAGTCGCT
Col1(M-R)	CTTGGTGGTTTTGTATTCGATGAC
GAPDH (H-F)	GGAGCGAGATCCCTCCAAAAT
GAPDH (H-R)	GGCTGTTGTCATACTTCTCATGG
ERN1 (H-F)	CACAGTGACGCTTCCTGAAAC
ERN1 (H-R)	GCCATCATTAGGATCTGGGAGA
XBP1s (H-F)	GCTGAGTCCGCAGCAGGTG
XBP1s (H-R)	GGGTCCAAGTTGTCCAGAATGC
PGRN (H-F)	AGGAGAACGCTACCACGGAC
PGRN (H-R)	GCTGACATTATCACAGGGGACAT
Aggrecan (H-F)	GTGCCTATCAGGACAAGGTCT
Aggrecan (H-R)	GATGCCTTTCACCACGACTTC
COL2 (H-F)	TGGACGATCAGGCGAAACC
COL2 (H-R)	GCTGCGGATGCTCTCAATCT
DCN (H-F)	ATGAAGGCCACTATCATCCTCC
DCN (H-R)	GTCGCGGTCATCAGGAACTT
ADAMTS5 (H-F)	GAACATCGACCAACTCTACTCCG
ADAMTS5 (H-R)	CAATGCCCACCGAACCATCT
MMP13 (H-F)	TCCTGATGTGGGTGAATACAATG
MMP13 (H-R)	GCCATCGTGAAGTCTGGTAAAAT
Ps:F:Forward R:Reverse M:Mouse H:Human	

Supplementary Table 3: Antibody and reagent information

**Antibody**	**Company**	**Catalog#**	**Dilution**
GAPDH	Affinity	AF7021	WB (1:4000)
β-actin	Abclonal	AC038	WB (1:10000)
PGRN(human)	Abcam	ab208777	WB (1:2000)
PGRN(human)	R&D	MAB2420	IF (1:200)
PGRN(mouse)	Abcam	ab187070	WB (1:1000), IF (1:200),IP (3μg), IHC (1:100)
IRE1α	CST	3294S	WB (1:1000), IF (1:200), IP (3μg)
IRE1α	Bioss	bs-16696R	IHC (1:200)
p-IRE1α	Affinity	AF7150	WB (1:1000), IF (1:200), IHC (1:200)
XBP1s	Biolegend	3294S	WB (1:1000), IF (1:200), IHC (1:50)
XBP1u/s	Affinity	AF5110	WB (1:1000)
Col2	Affinity	AF0135	WB (1:750), IHC (1:100)
Col2	Novus	NB600-844SS	IF (1:50)
Aggrecan	Affinity	DF7561	WB (1:1000), IHC (1:200)
COMP	Boster	A02443-1	IHC (1:200)
MMP13	Proteintech	18165-1-AP	IHC (1:200), IF (1:200)
ERK1/2	CST	4695	WB (1:2000)
p-ERK1/2	CST	4370	WB (1:2000), IHC(1:100)
U0126	MCE	HY-12031	
TNFα	Abcam	ab9642	
TNFR1 Ab	Santa	sc-8436	
TNFR2 Ab	Santa	sc-7862	

References:

1. Hao, Z. M., Yang, X., Cheng, X., Zhou, J. & Huang, C. F. Generation and characterization of chondrocyte specific Cre transgenic mice. *Yi Chuan Xue Bao* **29**, 424-9(2002).
2. Sakai, K. et al. Stage-and tissue-specific expression of a Col2a1-Cre fusion gene in transgenic mice. *Matrix Biology* **19**, 761-767(2001).
3. Kong, L. et al. Extracellular matrix protein 1, a direct targeting molecule of parathyroid hormone-related peptide, negatively regulates chondrogenesis and endochondral ossification via associating with progranulin growth factor. *Faseb J* **30**, 2741-2754(2016).
4. Almeida, S., Zhou, L. J. & Gao, F. B. Progranulin, a Glycoprotein Deficient in Frontotemporal Dementia, Is a Novel Substrate of Several Protein Disulfide Isomerase Family Proteins. *Plos One* **6**, (2011).
5. Glasson, S. S., Chambers, M. G., Van den Berg, W. B. & Little, C. B. The OARSI histopathology initiative - recommendations for histological assessments of osteoarthritis in the mouse. *Osteoarthritis Cartilage* **18**, S17-S23(2010).
6. KPH, P. et al. Osteoarthritis cartilage histopathology: grading and staging. *Osteoarthritis Cartilage* **14**, 13-29(2006).
7.Li, X. L. et al. Effect Of XBP1 Deficiency In Cartilage On The Regulatory Network Of LncRNA/circRNA-miRNA-mRNA. *International Journal of Biological Sciences* **18**, 315-330(2022).
